# A Natural Wish

**Published:** 1883-02

**Authors:** 


**A NATURAL WISH.**—It is very natural that we should wish the circulation of this JOURNAL largely extended. Has it ever occurred to our subscribers that it is their duty to do something toward this extension? If you have derived benefit from it, would it not be a kindly and neighborly act to induce your nearest friends to take it a year on trial? for it is very certain that by following its counsels, their health and happiness would be promoted and their lives extended for years.

HAVE YOU CHILDREN? You know how indifferent they are to your advice in too many cases; you know also that more importance is attached to what they see in print than to what you might say in regard to health. Would not the chances of their being benefited be greatly increased if you were to order the JOURNAL for each one of them? Their illness would be your greatest trouble, their death an irremediable grief; and yet thousands of lives are lost every year from inattention to the suggestions of one single article, to wit—about cooling off slowly after exercise.

